# Evaluation of the Secondary Transmission Pattern and Epidemic Prediction of COVID-19 in the Four Metropolitan Areas of China

**DOI:** 10.3389/fmed.2020.00171

**Published:** 2020-05-07

**Authors:** Longxiang Su, Na Hong, Xiang Zhou, Jie He, Yingying Ma, Huizhen Jiang, Lin Han, Fengxiang Chang, Guangliang Shan, Weiguo Zhu, Yun Long

**Affiliations:** ^1^Department of Critical Care Medicine, Peking Union Medical College Hospital, Peking Union Medical College, Chinese Academy of Medical Sciences, Beijing, China; ^2^Digital China Health Technologies Co. Ltd., Beijing, China; ^3^Department of Information Management, Peking Union Medical College Hospital, Peking Union Medical College, Chinese Academy of Medical Sciences, Beijing, China; ^4^Department of Epidemiology and Biostatistics, Institute of Basic Medicine Sciences, Peking Union Medical College, Chinese Academy of Medical Sciences, Beijing, China; ^5^Department of General Internal Medicine, Peking Union Medical College Hospital, Peking Union Medical College, Chinese Academy of Medical Sciences, Beijing, China

**Keywords:** COVID-19, novel coronavirus, secondary transmission, epidemic prediction, SEIR, basic reproduction number

## Abstract

Understanding the transmission dynamics of COVID-19 is crucial for evaluating its spread pattern, especially in metropolitan areas of China, as its spread could lead to secondary outbreaks. In addition, the experiences gained and lessons learned from China have the potential to provide evidence to support other metropolitan areas and large cities outside China with their emerging cases. We used data reported from January 24, 2020, to February 23, 2020, to fit a model of infection, estimate the likely number of infections in four high-risk metropolitan areas based on the number of cases reported, and increase the understanding of the COVID-19 spread pattern. Considering the effect of the official quarantine regulations and travel restrictions for China, which began January 23~24, 2020, we used the daily travel intensity index from the Baidu Maps app to roughly simulate the level of restrictions and estimate the proportion of the quarantined population. A group of SEIR model statistical parameters were estimated using Markov chain Monte Carlo (MCMC) methods and fitting on the basis of reported data. As a result, we estimated that the basic reproductive number, *R*_0_, was 2.91 in Beijing, 2.78 in Shanghai, 2.02 in Guangzhou, and 1.75 in Shenzhen based on the data from January 24, 2020, to February 23, 2020. In addition, we inferred the prediction results and compared the results of different levels of parameters. For example, in Beijing, the predicted peak number of cases was 467 with a peak time of March 01, 2020; however, if the city were to implement different levels (strict, moderate, or weak) of travel restrictions or regulation measures, the estimation results showed that the transmission dynamics would change and that the peak number of cases would differ by between 54% and 209%. We concluded that public health interventions would reduce the risk of the spread of COVID-19 and that more rigorous control and prevention measures would effectively contain its further spread, and awareness of prevention should be enhanced when businesses and social activities return to normal before the end of the epidemic. Further, the experiences gained and lessons learned from China offer the potential to provide evidence supporting other metropolitan areas and big cities with their emerging cases outside China.

## Introduction

The World Health Organization (WHO) named the virus “2019 novel coronavirus disease” (COVID-19) and the novel virus “severe acute respiratory syndrome coronavirus 2” (SARS-COV-2), which has attracted worldwide attention. The new coronavirus is a strain that has never been found in humans before. This virus can cause an acute respiratory disease, and common signs of infection include respiratory symptoms, fever, cough, shortness of breath, and dyspnea. In more severe cases, infection can cause pneumonia, severe acute respiratory syndrome, kidney failure, and even death (1).

According to WHO situation reports, the outbreak of COVID-19 has led to 79,407 confirmed cases worldwide and 2,622 deaths in 32 countries as of February 24, 2020, of which 64,287 were from Hubei, China. Numerous cases have been reported in other areas outside Hubei, including metropolitan areas of Beijing (*n* = 399) and Shanghai (*n* = 335) as well as other countries outside China, such as South Korea (*n* = 833), Japan (*n* = 144), and Italy (*n* = 124). With the continuously increasing number of cases, understanding the spread pattern of COVID-19 and monitoring spikes in the number of cases are crucial steps in providing evidence that could guide public health intervention strategies and healthcare policy making.

Several mathematical models and data analysis approaches attempting to estimate the transmission of COVID-19 have been recently reported (2–4). Public health interventions and transportation restriction effects for disease transmission have also been evaluated in some studies (5, 6). Some studies indicated that public intervention measures greatly mitigate the final size of the epidemic, and shift the turning point about 24 days before the turning point without these measures (7). Some noted that travel restrictions would not affect much unless combined with a 50% or higher reduction of transmission in the community (8). And a report from Imperial College COVID-19 Response Team concluded that the intensive intervention or something equivalently effective, such as combining home isolation of suspect cases, home quarantine of those living in the same household as suspect cases, and social distancing of the elderly and others at most risk of severe disease, could reduce transmission. However, this would need to be maintained until a vaccine becomes available, and the team also predicted that transmission will quickly rebound if interventions are relaxed, so it requires the combination of multiple interventions to have a substantial impact on transmission (9). In order to predict the outbreak size and time, researchers have published many different results for forecasting when the outbreak will peak in different areas (10, 11). These models are certainly useful to understand the emerging trends of COVID-19. However, there are several challenges to such timely analyses and forecasting. Due to barriers, such as the disease incubation period, asymptomatic infection, diagnosis testing capacity, overloaded medical staff, and complicated reporting processes, there can be delays or missed reporting in this evolving situation regarding the confirmation of cases. Furthermore, the adopted models have mostly been complicated with many pre-settings or assumptions or parameter values that are likely not accurate. Although some modeling approaches can estimate parameter values through statistical methods, they can only contribute a rough simulation for the modeling. As a result, those studies achieved different prediction results by using different methods and datasets.

To achieve a relatively objective judgment, given that that this new disease and complicated situation has many unknown factors, we used mathematical modeling methods to characterize COVID-19 transmission and used multiple datasets for ensuring the data reliability. Since individual data sources may be biased or incomplete, according to related studies, the use of multiple data sources rather than a single dataset can enable a more robust estimation of the underlying dynamics of transmission (12). Therefore, we investigated and collected data from four sources, including released data and official daily reports from commercial technology companies, academic institutes, authorities, or local healthcare commissions, and the World Health Organization, to minimize the resulting errors caused by potentially biased single data sources. The data were obtained from the Beijing Municipal Health Commission (BMHC) (13), Shanghai Municipal Health and Family Planning Commission (SMHFPC) (14), Health Commission of Guangdong Province (15), National Bureau of Statistics of China (NBSC) (16), Baidu Migration Big Data Platform (BMBDP) (17), Center for Systems Science and Engineering (CSSE) of Johns Hopkins University (18), and WHO coronavirus disease (COVID-2019) situation reports (19). Considering that the cases detected in these four cities were all imported or secondary transmission cases, and based on the reported data available after January 20, 2020, Chinese authorities have implemented prevention measures in these cities to contain the outbreak and prevent the disease from spreading; thus, we considered the secondary transmission pattern of COVID-2019 to be different than the early spread pattern in Wuhan, where the virus was rampantly transmitted without any prevention measures. Therefore, we collected data from January 24, 2020 (Chinese New Year's Eve) to February 23, 2020 to give an overall objective estimation of COVID-19 development in four high-risk metropolitan areas of China: Beijing, Shanghai, Guangzhou, and Shenzhen. We estimated how COVID-19 human-to-human transmission occurred in these large cities, which have developed considerable cases. We further used these estimates to forecast the potential risks and development trends of these four metropolitan areas inside China.

## Methods

To evaluate the COVID-19 spread pattern and estimate its transmission in four metropolitan areas, we used an adjusted SEIR model with data. We only considered human-to-human transmission in our models.

### Adjusted SEIR Model for COVID-19

**Figure 1:**
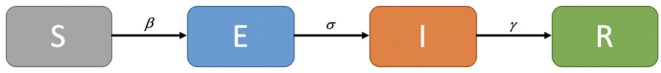
SEIR model.

The SEIR model is a deterministic metapopulation transmission model in which the population is divided into four classes: S (susceptible, people who are likely to be infected), E (exposed, people who are exposed), I (infectious, people who are infected), and R (removed, recovered and dead persons). We assumed that the epidemic risk started with infectious cases on February 3, 2020, when authorities announced that people were returning to work after the Chinese Spring Festival holiday. Therefore, we modeled a period beginning on February 3, 2020. The SEIR model state transition is shown in Figure 1. In our estimation, the entire population was initially susceptible since COVID-19 is an emerging new infectious disease and not all people have immunity against it. In January (before Chinese New Year), there were an estimated 3.246, 2.847, 3.430, and 3.271 million people flown out from Beijing, Shanghai, Guangzhou, and Shenzhen, respectively. We took this outflow number out from these four cities' initial populations and assume they returned after Chinese New Year by February 17, 2020. We estimated the initial exposed population using the number of confirmed cases during the next 7 days. We assumed that the median incubation period was 5–6 days (ranging from 0–14 days) based on the WHO report (20).

Based on the basic SEIR model, we further considered the influence of multiple factors on the transmission pattern as the situation unfolded, including public health intervention measures, people's self-protection behaviors, the diagnosis rate, population flow, etc.

Assuming that public health interventions contributed to the control of the dynamics of the epidemic, we incorporated a parameter that indicates the changes in the population flow into the model. According to the inflow index, outflow index, and urban daily adjusted index of the travel intensity from the Baidu Migration Big Data Platform, for the period from January 24, 2020 to February 23, 2020, we inferred that people's activity was obviously lower than the normal level for the same period last year. Furthermore, considering the Spring Festival population flow and those returning to work after the holiday (officially announced as February 3, 2020), we regarded that the risk for these four metropolitan areas grows with the inflow population increase starting on February 3, 2020, and the four cities executed 14 days quarantine policy for incoming travelers during that time, the spread was contained strictly, so an average introduced number of cases were counted into the model.

**Figure 2:**
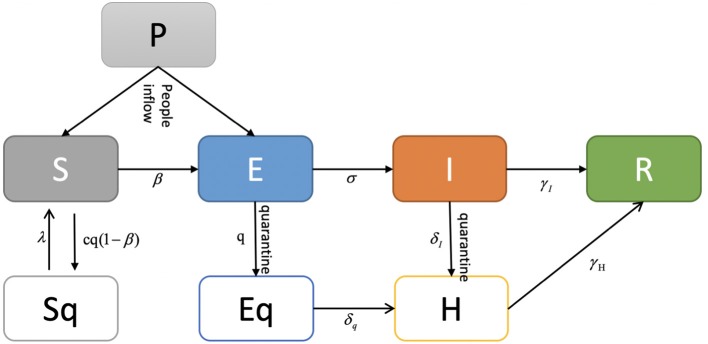
Adjusted SEIR model for COVID-19.

We also estimated the parameter values within these cities using the MCMC method. Cases in the reported data and other sources reported between January 24, 2020 and February 23, 2020 were used to adjust the model. Considering the possible complex influencing factors, we proposed an adjusted SEIR model for COVID-19 estimation, as displayed in Figure 2.

In the adjusted SEIR model, we considered the inflow of the city's population, so the total number of people was not fixed, and the population was divided into seven classes: *S* (susceptible, people who are likely to be infected), *E* (exposed, people who are exposed), *I* (infectious, people who are infected), *R* (recovered and dead persons), *Sq* (quarantined susceptible persons), *Eq* (isolated exposed persons), and *Iq* (isolated infected persons). The transmission dynamics are governed by the following equations:

$$\begin{array}{l} \{\begin{array}{l} \frac{dS}{dt}=-\left[c\beta +cq\left(1-\beta \right)\right]S\left(I+\theta E\right)+\lambda S_{q},\\ \frac{dE}{dt}=c\beta \left(1-q\right)S\left(I+\theta E\right)-\sigma E,\\ \frac{dI}{dt}=\sigma E-\left(\delta_{I}+\alpha +\gamma_{I}\right)I+i\\ \frac{dS_{q}}{dt}=\left(1-\beta \right)cqS\left(I+\theta E\right)-\lambda S_{q},\\ \frac{dE_{q}}{dt}=\beta cqS\left(I+\theta E\right)-\delta_{q}E_{q},\\ \frac{dH}{dt}=\delta_{I}I+\delta_{q}E_{q}-(\alpha +\gamma_{H})H,\\ \frac{dR}{dt}=\gamma_{I}I+\gamma_{H}H \end{array} \end{array}$$

where *q* is the quarantined proportion of exposed individuals, β is the transmission probability per contact, *c* is the contact rate which defines how many people are contacted with an infected person per day, and i is the estimated infected people within the inflow population each day. The quarantined infected people moved to the compartment *E*_*q*_ at a rate of βcq, while the quarantined uninfected people moved to the compartment *S*_*q*_ at a rate of (1–β)cq. Those who were not quarantined, if infected, moved to compartment *E* at a rate of β*c*(1 − *q*). θ is the transmission capability between the latent and the infected population. According to the reported results of related work, the transmission capability of the people in the incubation period and the diagnosed infected patients are similar (21), we assume that θ = 1. λ is the transition rate from the quarantined to susceptible population, σ is the transition rate from the exposed to the infected population, α is the mortality rate, δ_*I*_ is the transition rate from the infected population to the quarantined infected population, and γ_*I*_ is the recovery rate of the infected population. δ_*q*_ is the transition rate from the quarantined exposed population to the quarantined infected population, and γ_*H*_ is the recovery rate of the quarantined infected population.

### Parameter Estimate Methods

The MCMC method is a commonly used algorithm in modern statistical calculations. This algorithm provides an effective tool for establishing statistical models and is widely used in Bayesian calculations of complex statistical models (22). We used the MCMC method and Metropolis-Hastings(MH) algorithm sampling (23) with a normal distribution as the recommended distribution, estimated the parameters of the modified SEIR model to obtain the baseline estimation of parameters, incorporated the data collected from infectious disease reports into the above statistical inference, and simulated the process of infectious disease transmission to further fix some parameters on the basis of fitting reported data. Using Beijing as an example, the parameter estimates and initial values of the SEIR model are listed in Table 1.

**Table 1:** Parameters and initial values for the adjusted SEIR model (Beijing).

**Parameter**	**Referenced value**	**Methods**
c	5.4	MCMC and data fitting
β	2.18e-9	MCMC and data fitting
q	3.4e-5	MCMC and data fitting
σ	1/6	Source: WHO
λ	1/14	Source: NHC
δI	0.13	MCMC and data fitting
δq	0.13	MCMC and data fitting
γ_1_	0.0046	MCMC and data fitting
γ_H_	0.0092	MCMC and data fitting
α	0.2%	Source: WHO (2–20 report) (24)

In addition, to simulate the contact rate for model estimation, we used urban travel index data from Baidu, a major internet company in China that hosts the popular navigator app Baidu Maps, which indirectly monitors the real-time urban travel intensity and population flow. The Baidu index of travel intensity and population flow was converted into the corresponding coefficients for the contact rate and the quarantined susceptible population. In terms of the Baidu index, we simulated people's activity level by comparing our observed period (under strict interventions) with a normal level in the same period last year. We also consider the assumption scenario that when people return back to work (limited interventions), accordingly, we added the coefficients (0.6c, 0.8c, c, 1.5c, 2c) for the baseline contact rate to compare different effectiveness of interventions. Similarly, the coefficients were added to baseline quarantine proportion (0.6q, 0.8q, q, 1.5q, 2q).

### Basic Reproduction Number *R*_0_ Estimates

At the onset, when all people are susceptible, R_0_ is defined as the average number of new infections directly caused by a case in a population of people who are all susceptible. Given the model structure includes quarantine and isolation, we used the next generation matrix to derive a formula for the basic reproduction number after public health interventions were executed, the principal eigenvalue of the next generation matrix is the expectation of population growth and the equation is as follows and the parameter definition is same with adjusted SEIR model.

$$\begin{array}{l} R_{0}=\left[\frac{\beta c\left(1-q\right)}{\delta_{I}+\alpha +\gamma_{I}}+\frac{\beta c\theta \left(1-q\right)}{\sigma }\right]S_{0} \end{array}$$

## Results

### Data Characterization

**Figure 3:**
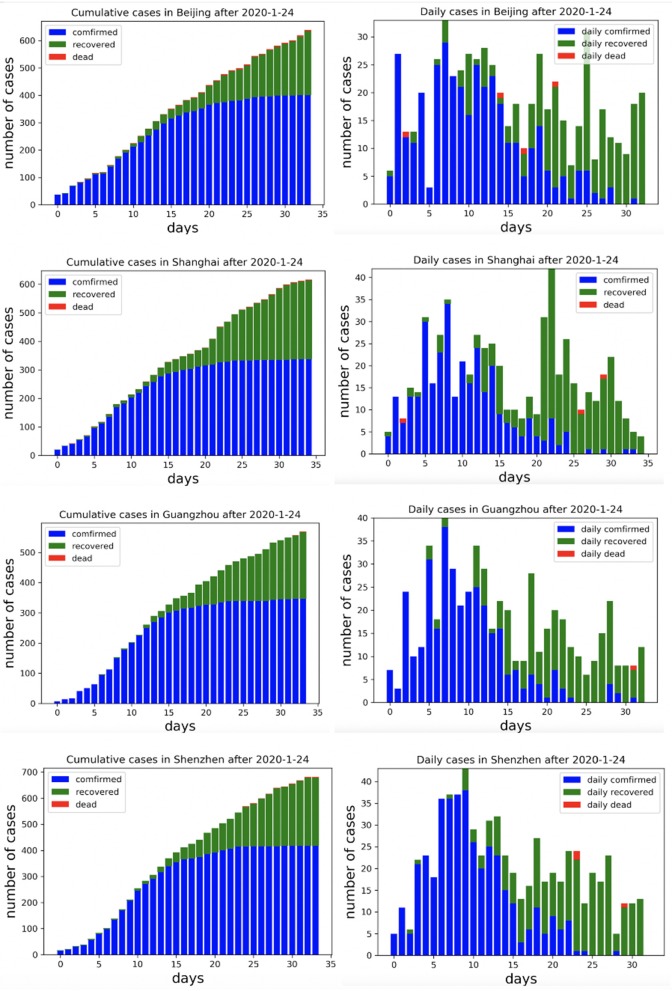
Cumulative and daily reported cases in four metropolitan areas in China.

To characterize the overall epidemic size and dynamics, Figure 3 shows the epidemic curve of COVID-19 cases identified in Beijing, Shanghai, Guangzhou, and Shenzhen from January 24, 2020 to February 23, 2020.

### Adjusted SEIR Model Estimation

**Figure 4:**
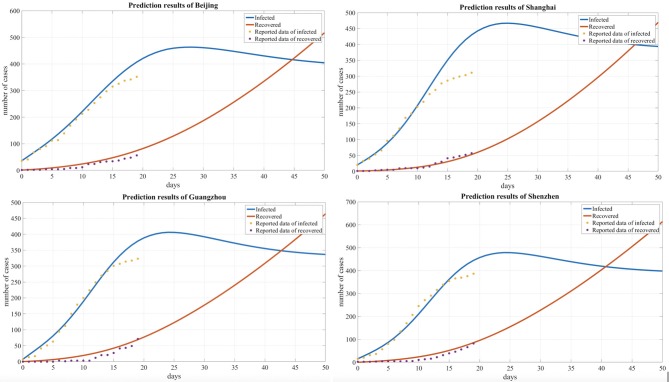
Comparison of the predicted and reported numbers of infected and recovered people for four cities.

We summarized and interpreted the transmission dynamics of COVID-19 in the four metropolitan areas. The adjusted SEIR model was used to predict cases in Beijing, Shanghai, Guangzhou, and Shenzhen, and Figure 4 shows the comparisons between the predicted results and actual results. The results are based on an assumption of no further imported cases to these cities since China implemented strong regulation measures during the observation period.

**Figure 5:**
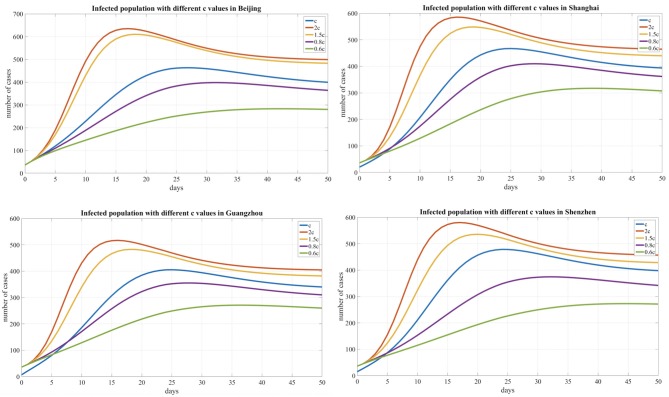
Infected population curves with different contact rates for four cities.

Based on our observations from the data shown in Table 2 and Figure 5 below, we also found that the number of infected individuals changed with different levels of public health interventions and that strict interventions could decrease the peak number of infected individuals compared with the scenario of weak interventions; accordingly, we used different contact rates to reflect the different levels of interventions. The baseline contact rate was derived by the MCMC method, and the results show that reducing the contact rate either persistently decreased the peak value or could delay the peak. In addition, with strict public intervention, the number of infected individuals eventually decreased, and the peak appeared sooner than it would with weak intervention methods. After February 3, 2020, as people returned to work after a holiday, many people returned to these cities, which was inferred from the Baidu transportation index. We added this information into the risk factors for the contact rate (1.5c, 2c). Accordingly, the number of infected individuals increased compared with the scenario of a decreased contact rate (0.8c, 0.6c).

**Table 2:** The effects of the contact rate on the peak time and peak value with an estimated value.

**Areas**	**Parameter c**	**2c**	**1.5c**	**C**	**0.8c**	**0.6c**
Beijing	Days to peak	16	19	27	31	41
	Peak value	642	608	476	399	286
Shanghai	Days to peak	16	19	25	29	40
	Peak value	592	545	473	406	309
Guangzhou	Days to peak	16	18	25	28	40
	Peak value	515	481	403	353	279
Shenzhen	Days to peak	17	20	25	32	45
	Peak value	688	542	487	478	377

**Figure 6:**
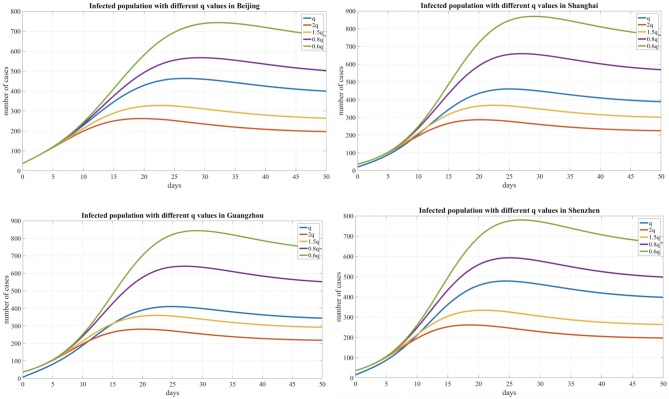
Infected population curve with different quarantined proportion of exposed individuals for four cities.

In addition, we compared the transmission dynamics with different quarantined proportion of exposed individuals, As shown in Table 3 and Figure 6, which reflects the contact tracing capability and management efforts of local governments, and the results show that reducing the quarantined proportion of exposed individuals (0.8q, 0.6q) led to an increase in the peak value and delayed the peak time. Conversely, the peak value decreased and an earlier peak time occurred with a higher quarantined proportion of exposed individuals (2q, 1.5q).

**Table 3:** The effects of the quarantined rate of exposed individuals on the peak time and peak value.

**Areas**	**Parameter q**	**2q**	**1.5q**	**Q**	**0.8q**	**0.6q**
Beijing	Days to peak	20	22	27	29	32
	Peak value	259	325	476	576	742
Shanghai	Days to peak	20	22	25	27	29
	Peak value	290	378	473	662	886
Guangzhou	Days to peak	20	23	25	27	29
	Peak value	389	352	403	643	842
Shenzhen	Days to peak	18	21	25	25	27
	Peak value	272	329	487	598	789

### *R*_0_ Estimation Results

We used the MCMC method to fit the model and adopted an adaptive MH algorithm to carry out the MCMC procedure. As a result, we inferred *R*_0_ = 2.91, 2.78, 2.02, and 1.75 for Beijing, Shanghai, Guangzhou, and Shenzhen, respectively.

## Discussions

Our analysis results strongly demonstrate that reducing secondary infections among close contacts would effectively limit human-to-human transmission, and public health measures, such as the rapid identification of cases, tracing and following up with people who had contact with an infected person, infection prevention and control in health care settings, and the implementation of health measures for travelers, can greatly prevent further spread of the disease.

The documented COVID-19 reproduction numbers range from 2.0 to 4.9 (6, 11, 25), which are based on cases that developed during different transmission phases and in different areas. For instance, the *R*_0_ in Wuhan was obviously higher than that in other cities during the timeframe analyzed. Furthermore, after implementing the prevention measures employed by the Chinese government and local authorities, we regarded the inferred *R*_0_ results of the four cities as reasonable and interpretable.

In this study, we aimed to monitor COVID-19 trends after cases were imported into other cities and estimate the spread pattern by mathematical modeling, which can be helpful for evaluating the potential risk and severity of new outbreaks. The results of our study show that, for four metropolitan areas of China, the containment measures were an effective control at that time; however, it is imperative to raise awareness in the population and prevent potential outbreak risks going forward. The study has limitations. The present reported data are insufficient to understand the full epidemiological pattern of COVID-19 transmission and new potential outbreaks. For example, the estimates in this manuscript have a certain degree of uncertainty and delays due to the limitations in reporting mechanisms over the course of the natural history of the cases, the impact of other potential asymptomatic cases, and some unreported cases. Some studies were conducted with the assumption that a small fraction, 20%, were not reported (7) and others reported the estimated asymptomatic proportion was 17.9% (26) or 60% (21). Evidently, such asymptomatic infectious cases are not fully reported by current testing method. However, some studies suggested crowdsourced data could be compiled and analyzed as an complementation of officially released data, which could perhaps help in improving the analysis results (27–29).

As concluded from the WHO-China Joint Mission report (30), the COVID-19 transmission dynamics are inherently contextual, as are the dynamics for any outbreak, and people worldwide need to work together to defend against this disease. To do this, it is necessary to: (1) enhance the understanding of the evolving COVID-19 and the nature and the impact of ongoing containment measures; (2) share knowledge on the COVID-19 response and preparedness measures being implemented in countries affected by or at risk of importations of COVID-19; (3) generate recommendations for adjusting COVID-19 containment and response measures in China and internationally; and (4) establish priorities for a collaborative program of work, research, and development to address critical gaps in knowledge, responses, readiness tools, and strategies.

As a consequence of our study, we concluded that the outbreak could be greatly reduced by strict public health interventions. The public intervention strategies and implemented protection measures conducted in these four areas may help provide epidemiological suggestions to governments that guide measures for the international cases that are rapidly emerging.

## Data Availability Statement

The datasets analyzed in this article are not publicly available because they are kept by a private affiliation (Peking Union Medical College Hospital). Requests to access the datasets should be directed to Na Hong (hongna@dchealth.com) or Yun Long (ly_icu@aliyun.com).

## Author Contributions

LS, NH, and XZ contributed equally. WZ, YL, and GS take responsibility for the integrity of the work as a whole, from inception to published article. NH and LS were responsible for study design and conception and drafted the manuscript. FC and LH collected and cleaned the data. JH, YM, and HJ were responsible for data modeling and analysis. WZ, GS, and XZ interpreted the results. LS, NH, and XZ drafted the manuscript. All authors revised the manuscript for important intellectual content.

## Conflict of Interest

NH, JH, YM, LH, and FC were employed by the company Digital China Health Technologies Co. Ltd., Beijing, China. The remaining authors declare that the research was conducted in the absence of any commercial or financial relationships that could be construed as a potential conflict of interest.

## References

[1] Coronavirus. World Health Organization (2020). Available online at: https://www.who.int/health-topics/coronavirus (accessed Febraury 25, 2020).

[2] Hermanowicz SW Forecasting the Wuhan coronavirus (2019-nCoV) epidemics using a simple (simplistic) model. medRxiv. (2020). 10.1101/2020.02.04.20020461

[3] Imai N Dorigatti I Cori A Donnelly C Riley S Ferguson NM Report 2: Estimating the Potential Total Number of Novel Coronavirus Cases in Wuhan City, China. Imperial College London (2020).

[4] Liu T Hu J Kang M Lin L Zhong H Xiao J Transmission Dynamics of 2019 Novel Coronavirus (2019-nCoV). The Lancet. (2020). 10.2139/ssrn.3526307

[5] Tang B Wang X Li Q Bragazzi NL Tang S Xiao Y. Estimation of the transmission risk of the 2019-nCoV and its implication for public health interventions. J Clin Med. (2020) 9:462. 10.3390/jcm9020462 32046137 PMC7074281

[6] Zhao S Lin Q Ran J Musa SS Yang G Wang W. Preliminary estimation of the basic reproduction number of novel coronavirus (2019-nCoV) in China, from 2019 to 2020: A data-driven analysis in the early phase of the outbreak. Int J Infect Dis. (2020) 92:214–7. 10.1016/j.ijid.2020.01.050 32007643 PMC7110798

[7] Liu Z Magal P Seydi O Webb G. Understanding unreported cases in the COVID-19 epidemic outbreak in Wuhan, China, and the importance of major public health interventions. Biology. (2020) 9:50. 10.3390/biology9030050 32182724 PMC7150940

[8] Chinazzi M Davis JT Ajelli M Gioannini C Litvinova M Merler S. The effect of travel restrictions on the spread of the 2019 novel coronavirus (COVID-19) outbreak. Science. (2020) eaba9757. 10.1126/science.aba9757 PMC7164386 32144116

[9] Ferguson NM Laydon D Nedjati-Gilani G Imai N Ainslie K Baguelin M Impact of Non-pharmaceutical Interventions (NPIs) to Reduce COVID-19 Mortality and Healthcare Demand. London: Imperial College (2020). 10.25561/77482 PMC7140590 32270376

[10] Read JM Bridgen JR Cummings DA Ho A Jewell CP Novel coronavirus 2019-nCoV: early estimation of epidemiological parameters and epidemic predictions. medRxiv. (2020). 10.1101/2020.01.23.20018549 PMC8165596 34053269

[11] Wu JT Leung K Leung GM. Nowcasting and forecasting the potential domestic and international spread of the 2019-nCoV outbreak originating in Wuhan, China: a modelling study. Lancet. (2020) 395:689–97. 10.1016/S0140-6736(20)30260-9 32014114 PMC7159271

[12] Funk S Eggo RM. Early dynamics of transmission and control of COVID-19: a mathematical modelling study. Lancet Infect Dis. (2020). 10.1016/S1473-3099(20)30144-4. [Epub ahead of print]. PMC7158569 32171059

[13] Decker C The past, present, and future of clinical data standards. In: SAS Global Forum 2010 Conference. Seattle, WA (2010). p. 14.

[14] Shanghai Municipal Health and Family Planning Commission. (2020). Available online at: http://wsjkw.sh.gov.cn/yqfk2020/ (accessed Febraury 25, 2020).

[15] Health Commission of Guangdong Province. (2020). Available online at: http://wsjkw.gd.gov.cn/zwyw_yqxx/ (accessed Febraury 25, 2020).

[16] National Bureau of Statistics of China. (2020). Available online at: http://data.stats.gov.cn/ (accessed Febraury 25, 2020).

[17] Baidu Migration Big Data Platform. (2020). Available online at: http://qianxi.baidu.com/ (accessed Febraury 25, 2020).

[18] Dong E Du H Gardner L. An interactive web-based dashboard to track COVID-19 in real time. Lancet Infect Dis. (2020). 10.1016/S1473-3099(20)30120-1. [Epub ahead of print]. PMC7159018 32087114

[19] Organization WH Coronavirus Disease (COVID-2019) Situation Reports. World Health Organization (2020). Available online at: https://www.who.int/emergencies/diseases/novel-coronavirus-2019/situation-reports (accessed Febraury 25, 2020).

[20] Organization WH Coronavirus Disease 2019 (COVID-19) Situation Report – 30. World Health Organization (2020). Available onlie at: https://www.who.int/docs/default-source/coronaviruse/situation-reports/20200219-sitrep-30-covid-19.pdf (accessed Febraury 25, 2020).

[21] Qiu J. Covert coronavirus infections could be seeding new outbreaks. Nature. (2020). 10.1038/d41586-020-00822-x. [Epub ahead of print]. 32203376

[22] Endo A Van Leeuwen E Baguelin M. Introduction to particle Markov-chain Monte Carlo for disease dynamics modellers. Epidemics. (2019) 29:100363. 10.1016/j.epidem.2019.100363 31587877

[23] Hastings WK Monte Carlo sampling methods using Markov chains and their applications. Biometrika. (1970). 57:97–109. 10.2307/2334940

[24] Organization WH Coronavirus disease 2019 (COVID-19) Situation Report – 31. World Health Organization (2020). Available online at: https://www.who.int/docs/default-source/coronaviruse/situation-reports/20200220-sitrep-31-covid-19.pdf (accessed Febraury 25, 2020).

[25] Shen M Peng Z Xiao Y Zhang L Modelling the epidemic trend of the 2019 novel coronavirus outbreak in China. bioRxiv. (2020). 10.1101/2020.01.23.916726 PMC7831648 33521762

[26] Mizumoto K Kagaya K Zarebski A Chowell G. Estimating the asymptomatic proportion of coronavirus disease 2019 (COVID-19) cases on board the Diamond Princess cruise ship, Yokohama, Japan. Eurosurveillance. (2020) 25:2000180. 10.2807/1560-7917.ES.2020.25.10.2000180 32183930 PMC7078829

[27] Arbia G A note on early epidemiological analysis of coronavirus disease 2019 outbreak using crowdsourced data. arXiv preprint arXiv: 2003.06207. (2020).

[28] Leung GM Leung K Crowdsourcing data to mitigate epidemics. Lancet Digital Health. (2020) 2:E126–7. 10.1016/S2589-7500(20)30055-8 PMC7158995 32296776

[29] Sun K Chen J Viboud C. Early epidemiological analysis of the coronavirus disease 2019 outbreak based on crowdsourced data: a population-level observational study. Lancet Digital Health. (2020) 2:E201–8. 10.1016/S2589-7500(20)30026-1 32309796 PMC7158945

[30] Organization WH Report of the WHO-China Joint Mission on Coronavirus Disease 2019 (COVID-19). (2020). Available online at: https://www.who.int/docs/default-source/coronaviruse/who-china-joint-mission-on-covid-19-final-report.pdf (accessed Febraury 25, 2020).

